# Prediction of Potential Ionic Liquids (ILs) for the Solid–Liquid Extraction of Docosahexaenoic Acid (DHA) from Microalgae Using COSMO-RS Screening Model

**DOI:** 10.3390/biom10081149

**Published:** 2020-08-06

**Authors:** Shiva Rezaei Motlagh, Razif Harun, Dayang Radiah Awang Biak, Siti Aslina Hussain, Amal A. Elgharbawy, Ramin Khezri, Cecilia Devi Wilfred

**Affiliations:** 1 Department of Chemical and Environmental Engineering, Faculty of Engineering, University Putra Malaysia, Serdang 43400 UPM, Selangor, Malaysia; shiva.rezaei.m@gmail.com (S.R.M.); dradiah@upm.edu.my (D.R.A.B.); aslina@upm.edu.my (S.A.H.); 2 International Institute for Halal Research and Training (INHART), International Islamic University Malaysia, Gombak, Kuala Lumpur 50728, Malaysia; amal.elgharbawy@gmail.com; 3 Department of Chemical Engineering, Faculty of Engineering, Chulalongkorn University, Bangkok 10330, Thailand; ramin.khezri@gmail.com; 4 Department of Fundamental and Applied Sciences, Centre of Research in Ionic Liquids (CORIL), Universiti Teknologi Petronas, Bandar Seri Iskandar 32610 UTP, Perak, Malaysia; cecili@utp.edu.my

**Keywords:** DHA, omega-3, ionic liquids, COSMO-RS, extraction, infinite dilution capacity value

## Abstract

This study performs a screening of potential Ionic Liquids (ILs) for the extraction of Docosahexaenoic Acid (DHA) compounds by the calculation of capacity values. For this purpose, a Conductor-Like Screening Model for Real Solvents (COSMO-RS) was employed to study the molecular structures of the ILs, and therefore, predict their extraction potential. The capacity values of 22 anions combined with 16 cations based ILs, were investigated to evaluate the effectiveness of ILs in the extraction of DHA. It was found that among the investigated ILs, a combination of tetramethyl ammonium with SO_4_ or Cl was the best fit for DHA extraction, followed by pyrrolidinium, imidazolium, pyridinium and piperidinium. Furthermore, it was observed that the extraction capacity and the selectivity of ILs decreased with an increase in alkyl chain length; therefore, ethyl chain-ILs, with the shortest chain lengths, were found to be most suitable for DHA extraction. The predicted results were validated through the experimentally calculated extraction yield of a DHA compound from *Nannochloropsis sp.* Microalgae. Five selected ILs, namely [EMIM][Cl], [BMIM][Cl], [TMAm][Cl], [EMPyr][Br] and [EMPyrro][Br], were selected from COSMO-RS for empirical extraction purposes, and the validation results pinpointed the good prediction capabilities of COSMO-RS. The findings in this study can simplify the process of selecting suitable ILs for DHA extraction and reduce the number of required empirical evaluations.

## 1. Introduction

In nature, omega-3 polyunsaturated fatty acids (omega-3 PUFA) are typically found in three distinct forms of: eicosapentaenoic acid (EPA), docosahexaenoic acid (DHA) and alphalinolenic acids (ALA). In recent years, omega-3 PUFAs have attracted growing attention as vital compounds because of their various health and disease reduction advantages [1,2]. They have been used in pharmaceutical and nutraceutical products due to their biochemical functions [3]. Many studies have demonstrated their positive effects on health and disease prevention. The risks of cancer, cardiovascular disease or rheumatoid arthritis can be reduced by certain dietary modifications including consuming omega-3s [4]. Also, there is a strong evidence of the clinical benefits of this type of omega-3 PUFA in terms of improving neurological function and alleviating mental disorders [5]. 

The consumption of 0.9 g per day of omega-3 PUFA for the general population was recommended by Food Standard Agency (FSA) in 2007 [6]. However, these highly unsaturated fatty acids, while considered essential, cannot be synthesized in the human body due to the absence of Δ−15 and Δ−12 desaturated enzymes [7]. Therefore, PUFA must be taken through the diet and supplements [8]. There are several sources of EPA and DHA, including fish, plants and fungi, but the quantity of both compounds in microalgae is comparatively greater [9]. Furthermore, microalgae provides the human body with countless health advantages [10]. Typically, lipid extraction from microalgae is achieved by standard techniques such as Soxhlet and Bligh and Dyer using hexane, chloroform and other organic solvents [11]. However, there are a number of challenges associated with the use of those solvents, e.g., create health, security and handling issues. As highlighted by [12], organic solvents require effective recovery for commercialization. Moreover, most organic solvents tend to contribute to air pollution due to their relatively high volatility. Additionally, they are often toxic, flammable and relatively expensive, and certain compounds may not work well due to their low solubility. In the case of microalgae, lipids are typically trapped inside the cell walls which also contain proteins, i.e., heavy and complex polysaccharide compounds, that make them chemically resistant to nonpolar solvents [13]. In order to extract the lipids, hexane is required to cross the polar phospholipid membrane (i.e., the protein band); this process is complicated. However, polar solvents such as methanol and chloroform, which are usually used in the Bligh and Dyer method, are capable of crossing the phospholipid membrane via diffusion, and hence, extracting the lipids [14].

With regards to the aforementioned drawbacks of using organic solvents, ionic liquids (ILs) are suitable alternatives which has been recommended in several studies and by many researchers [15,16,17]. The use of ILs for solvent extraction provides a number of benefits, thanks to their remarkable properties such as low vapor pressure, high solubility and adequate thermal stability, making them suitable for the containment, recycling and regeneration of products. In addition, ILs involve less energy than organic solvents because of their unique features which facilitate the extraction process by decreasing its complexity. ILs are also involved with much simpler solvent regeneration [18]. However, in selecting ILs as solvents, it must be considered that their stability is strongly influenced by the level of moisture, as well as their miscibility with molecular liquids. Other drawbacks are related to their high cost, as well as environmental concerns regarding their disposal, particularly in the large-scale pretreatment of lignocellulosic biomasses. However, both of these problems can be addressed thanks to the high recyclability of ILs [19]. The correct choice of an anion–cation combination is essential, as it determines the physicochemical characteristics of ILs, e.g., density, viscosity, melting point, water miscibility, polarity, pH and coordination capability [20]. Compared to conventional solvents, ILs present better performance with higher extraction yields; however, the high cost of using ILs has resulted in limited number of studied cases, despite the number of known, potentially suitable compounds (10^18^) [21]. Screening tools that use theoretical and computational models to estimate the thermodynamics of ILs are valuable because they can be used to select anion–cation combinations properly, and thus, reduce the time, resources and overall cost of experiments for a large number of ILs [22]. The appropriate choice of ILs can influence the cost of heating, cooling, materials used and other process technology-related factors, as well as the product quality and the environmental and safety aspects of the extraction method; therefore, it is essential to predict the characteristics of the ILs before beginning experiments [23,24]. 

Although considered green solvents, it is not yet understood how ILs interact with other solutes; this could be determined by calculating the capacity values. The activity coefficient value at infinite dilution (AC^∞^) calculated as a function of temperature is an indicator of whether a specific solvent has adequate power to be used in extraction [25,26,27]. In the case of a solute in the ILs, the AC^∞^ is a key factor to apply in the preliminary selection of solvents for the purpose of extraction [28,29,30]. The behavior of a dissolved compound which is bounded by the molecules of a solvent can be explained by AC^∞^. Furthermore, AC^∞^ can be utilized directly to select a suitable solvent for extraction, in addition to having direct, crucial, practical uses for industrial-scale processes [25,31,32,33,34]. 

This study defines the activity coefficient as the effective concentration of a solute in a solvent whereby the activity of its substances determines the chemical potential of the solute. Once the solute concentration reaches values close to zero, it is referred to as the activity coefficient at infinite dilution [35]. Existing models for the prediction of AC^∞^ include UNIFAC, NRTL, Wilson or UNIQUAC and GCM. All of these models were developed based on the assumption that (to a great extent) the free energy of molecules in solution is additive. Although there are many advantages associated with the use of these predictive models, they cannot differentiate between isomers, and involve a huge amount of experimental information to derive the parameters of interaction between the subgroup and the group [27,36]. The UNIFAC prediction model has been observed to not work well with systems including strong hydrogen-bond networks like water. Moreover, UNIFAC may also have trouble with ILs, since it was parametrized by solely nonionic systems [37]. The original hypothesis for a specified group, according to GCMs, is that the energy interaction of any system as a whole can be determined by the inclusion of interaction energies from its functional groups. Therefore, GCMs are extremely dependent on the availability of group interactions and the associated parameters [30]. For these reasons, a liquid can be defined as a mixture of interacting structural groups, rather than a mixture of interacting molecules.

The Conductor-like Real Solvent Screening Model (COSMO-RS) was first implemented by [38] as a strategy based on computational quantum mechanics that can be used as an alternative instrument for predicting solvent thermos-physical characteristics and intermolecular interactions in solution [39]. In addition, COSMO-RS relies solely on the unimolecular quantity chemical calculations of the individual species in the scheme, as specified by [40,41]. Only a few input parameters are required to apply in COSMO-RS, and the parameters for each substance need to be estimated only once [42]. Several researcher have pointed the significance COSMO-RS as a tool to solve the thermodynamic and chemical engineering problems associated with activity coefficients [35]. A number of restrictions have been identified regarding the use of COSMO-RS, including the impossibility of applying high-temperature and high-pressure vapor–liquid balances, as well as polymer systems thermodynamics [43]. Aside from these restrictions, COSMO-RS has numerous benefits which are not limited to the calculation of the activity coefficient. The software can also predict the chemical potential, vapor pressure, solvent partition coefficient, solubility, enthalpy, Gibbs free energy and lots of other thermodynamic properties of a compound. Therefore, COSMO-RS can be considered a powerful prediction tool, especially for use with a large data bank of compounds where accessibility to experimental data is limited, or when performing a large number of experimentations is impractical [44].

There are many literature reports on the applicability of liquid–liquid extraction ILs using several performance indices such as ability, distribution coefficient, endless dilution and selectivity activity coefficient. In a previous study, COSMO-RS was used to predict the selectivity and distribution coefficient of ILs ([EMIM][EtSO_4_] and [EMIM][CH_3_COO]) for use as a solvent to extract benzothiophene from n-hexane. The greater selectivity for ethyl sulphate-based ILs was recorded and the outcome was a greater distribution coefficient for acetate-based ILs [37]. In another study, COSMO-RS was used to examine 264 ILs consisting of combinations of cations and anions to determine the most appropriate extracting solvent for the combination of benzene and cyclohexane. [OMIM][AlCl_4_] was selected as the strongest candidate compound [45]. Moreover, in a previous study, the potential of different types of ILs (diverse cation/anion combination) for EPA (C:20, n-3) compound extraction was investigated through the calculation of capacity values using the COSMO-RS prediction tool. As a result, the highest extraction capacity belonged to [TMAm][SO_4_]. Furthermore, it was shown that ILs with lower anion sizes have greater extraction capacities and load densities than those with bigger anions [46]. 

As far as the authors are aware, no work has been carried out on predicting the capacity values of ILs in analyzing the efficacy of extracting DHA compounds. Thus, the novelty of this study is the use of the COSMO-RS predictive method to screen the potential of ILs for DHA extraction. Hence, this research provides a good database for authors who are looking to extract long chain fatty acids using ILs as green solvents.

We performed a screening evaluation using the COSMO-RS method to determine the capacity values of 352 cations–anions combination base ILs in order to identify the most suitable types to apply to solid–liquid extraction of DHA from microalgae. The significance of this study is that through the reported screening methodology, one can determine the capacity values of numerous ILs as green solvents to apply to DHA extraction without having to perform a substantial number of expensive experiments. The most important aspect of this study is that it predicts the capacity values of a variety of ILs in solid–liquid extractions from microalgae biomasses, while most related research was focused on liquid–liquid extraction.

## 2. Materials and Methods

### 2.1. Computational Methods of COSMO-RS

**Figure 1:**
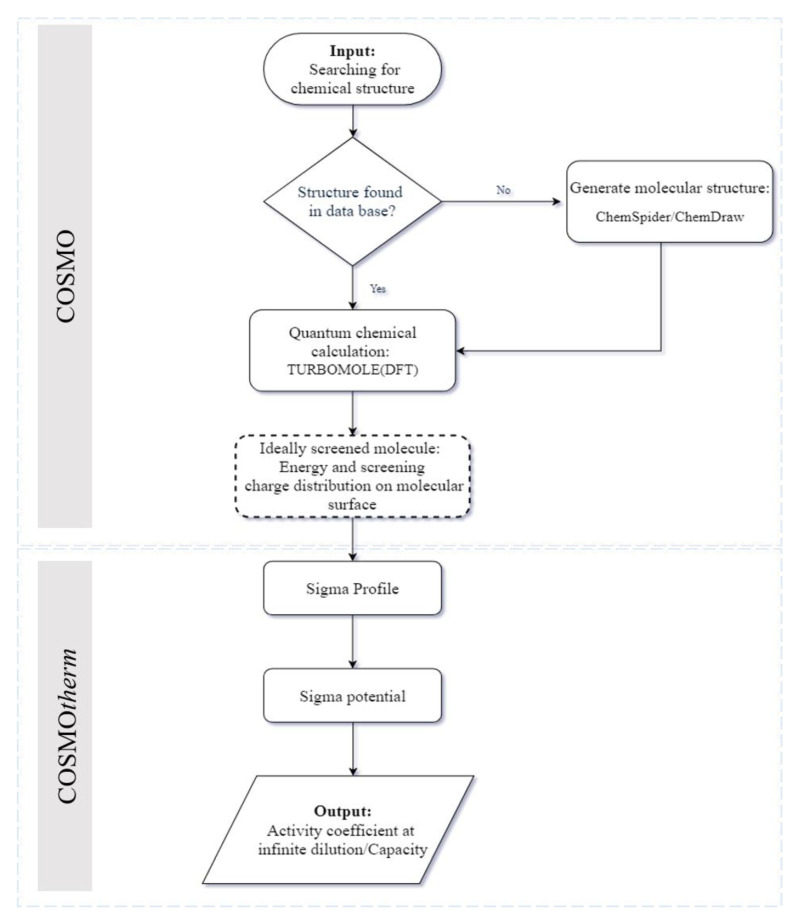
COSMO-RS calculation steps for solid–liquid extraction.

The protocol used for the calculation is schematically illustrated in Figure 1. In the first step, the structure of chemical species (solvent and solute) must be identified. For this purpose, the structure of each species was either looked up in the COSMO-RS data library or derived from a third-party source, like the ChemSpider software. COSMO-RS was subsequently used to optimize the chemical structures of interacting species and, as a consequence, to predict their related thermodynamic properties. In the next step, with the help of TURBOMOLE 6.1 package, the molecular geometry of DHA, as well as the corresponding charge density, was predicted and saved in a file format that is readable in COSMO platform [47]. The quantity calculations were subsequently made using Density Functional Theory (DFT) with identity resolution (RI) approximation. The Becke and Perdew function, with the triple-z valence polarized basis set BP_TZVP_C30_1301 version (COSMOlogic GmbH & Co KG, Leverkusen, Germany) was applied for energy calculation [48,49,50]. As the characteristics of ILs have been shown to be considerably dependent on their structures, the impact of the IL structure on DHA extraction should be explored. To this end, ILs consisting of different anion and cation combinations were studied. Solvent extraction is a simple and cost-effective method that is suitable for the extraction of DHA. 

The most important parameters to consider in solid–liquid processing are the infinite dilution capacity that accounts for a solvent’s extractive intensity. This research conducted a screening of a mixture of 16 cations with 22 anions to explore the impact of variations in anionic and cationic composition, and to select a mixture to enhance the capacity of ILs to extract DHA compounds. It is noteworthy that all averaged surface segments, according to the assumptions of COSMO-RS, are independent. Significant parameters linked to DHA’s net ground load, including the sigma-profile (σ-profile) and sigma-potential (σ-potential), were calculated. The σ-profile accounts for the charge density profiles of the molecular surface, whereas the σ-potential is related to determining the chemical characteristics of DHA. Equation (1) indicates the formulation of the sigma profile of the mixture $\left(p_{s}\left(\sigma \right)\right)$:

(1) $$p_{s}\left(\sigma \right)=\sum_{i=s}X_{i}\;p^{X_{i}}\left(\sigma \right)$$

where $X_{i}$ and $p^{X_{i}}\left(\sigma \right)$ in Equation (1) indicate the mole fraction and sigma profile of compound *X*, respectively.

When two interaction surface fragments (i.e., σ and $\sigma^{\prime }$ or $\sigma_{acceptor}$ and $\sigma_{donor}$) are placed on a hydrogen bond acceptor or donor atom, the generated polarization charges form two major kinds of molecular interaction energies, i.e., electrostatics energy ($E_{misfit}$) and hydrogen bonding energy ($E_{HB}$), and one less specific energy related to van der Waals interactions ($E_{vdW}$). Equations (2)–(4) can be used to calculate of the electrostatic energy ($E_{misfit}$), hydrogen bonding energy ($E_{HB}$) and van der Waals ($E_{vdW}$) interactions energy, respectively.

(2) $$E_{misfit}\left(\sigma ,\sigma^{\prime }\right)=a_{eff}\frac{\alpha^{\prime }}{2}{\left(\sigma +\sigma^{\prime }\right)}^{2}$$

(3) $$E_{HB}=a_{eff}C_{HB}\min\left(0;\sigma_{donor}+\sigma_{HB}\right)\max\left(0;\sigma_{acceptor}-\sigma_{HB}\right)$$

(4) $$E_{vdW}=a_{eff}\left(\tau_{vdW}+\tau_{vdW}^{\prime }\right)$$

where $\alpha^{\prime }$ is an interaction parameter, $a_{eff}$ is the effective average surface area that accounts for the size of a thermodynamically independent contact and $\mu_{s}\left(\sigma \right)$ is the sigma potential (distribution function) that describes the characteristic of a solvent or mixture with the level of its attraction to a surface area with polarity σ [51], as determined by Equation (5). Thus, the solvent’s molecular interactions are completely defined by $P_{s}\left(\sigma^{\prime }\right)$ and the surface segment’s chemical potential can be calculated by solving a combined set of nonlinear equations, i.e., Equations (2) and (3).

(5) $$\mu_{s}\left(\sigma \right)=-\frac{RT}{a_{eff}}ln\left[\int^{\;}P_{s}\left(\sigma^{\prime }\right)exp\left(\frac{a_{eff}}{RT}\left(\mu_{s}\left(\sigma^{\prime }\right)-E_{misfit}\left(\sigma ,\sigma^{\prime }\right)-E_{HB}\left(\sigma ,\sigma^{\prime }\right)\right)\right)d\sigma^{\prime }\right]$$

In Equation (5), R is ideal gas constant and T is absolute temperature. The solvent capacity values of ILs for the extraction of DHA are calculated by the following equation:

(6) $$C^{\infty }={\left(\frac{1}{AC^{\infty }}\right)}^{Ionic\;liquid\;phase}$$

where $AC^{\infty }$ is the activity coefficient of the solvent at infinite dilution which is a function of temperature [52]. As prospective solvents in DHA extraction, ILs with greater capability values were considered more desirable in this research.

### 2.2. Process Stages in Screening ILs

The data collection adds the DHA chemical structure to the COSMO-RS data bank, which uses TZVP to optimize and adjust the thermodynamic properties of the DHA. Table 1 demonstrates the chemical formula and structure of the DHA compound. ILs are then created from a combination of imidazolium, pyridinium, pyrrolidinium, piperidinium and tetramethyl ammonium cations with hydrophobic and hydrophilic anions. The cation- and anion-based ILs that were formed and used in current study are listed in Table 2. 

Furthermore, the activity coefficient and capacity value of each IL corresponding to the DHA compound were derived by the software and the sigma profile and sigma potential of DHA were calculated.

### 2.3. Material and Methods of Experimental Validation

The frozen dried biomass (*Nannochloropsis sp.*) was bought and transported from Longevity Superfoods (Utah, USA batch no: 32490). The powdered biomass was kept wrapped in its packaging and stored when not being used in experiments. Hexane, chloroform (>99.8%) and methanol were supplied by R&M Chemicals (Kuala lumpur, Malaysia) in analytical grade. The ionic liquids including 1-ethyl-3 methyl imidazolium chloride ([EMIM][Cl], 94.5%), tetramethyl ammonium chloride ([TMAm][Cl], ≥99%), 1-ethyl-1-methyl pyrrolidinium bromide ([EMPyrro][Br], 99%), 1-butyl-3-methyl imidazolium chloride ([BMIM][Cl], 98%) and 1-ethyl-3-methyl pyridinium bromide ([EMPyr][Br], 98%) were purchased from Merck (Kenilworth, NJ, USA) and utilized without any additional purification. The microwave oven reactor (Samsung, ME711K) was purchased in Malaysia.

### 2.4. Microwave-Assisted Extraction (MAE) of Lipids Using ILs and Prodcution of DHA via Transesterification

**Figure 2:**
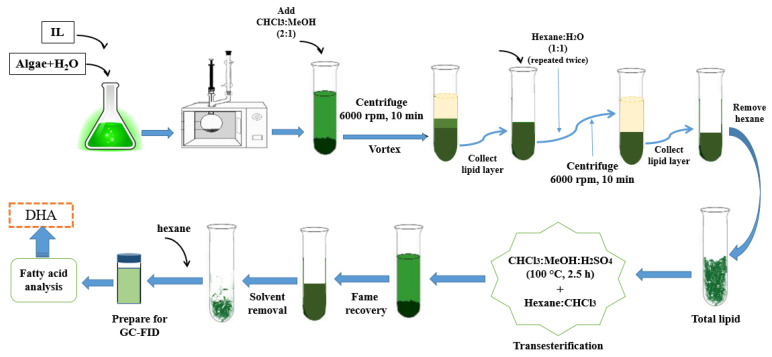
Schematic of experimental setup of lipid and DHA extraction from microalgae biomass.

MAE was employed to extract the total lipid from a microalgae *Nannochloropsis sp.* biomass at 700 W energy and a frequency of 2.45 GHz. The method was adapted from a previous work [53]. A solution consisting 2% *w*/*v* IL and 15 mL distilled water was prepared and used as the extraction medium for 0.5 g dry microalgae biomass (3.3% *w*/*v* biomass loading). Using a domestic microwave, the sample was heated to 90 °C for 25 min. Next, methanol and chloroform were added to the solution before it underwent centrifugal phase separation at 6000 rpm for 10 min. The sample formed two separate phases. The chloroform phase was then isolated and washed with a solution of distilled water and hexane, 2 or 3 times, to ensure that any residual polar compounds had been completely removed. Finally, lipids were recovered from the hexane phase through evaporation. In order to synthesize the DHA compounds, transesterification of the extracted lipid was performed. A method of producing fatty acid methyl esters (FAMEs) that contain DHA was adapted from previous literature reports [54,55]. The produced FAMEs were duly separated and sent for analysis via gas chromatography (Agilent 6890 GC, USA) equipped with a flame ionization detector (FID). The schematic sequences of empirical lipid extraction and DHA production are presented in Figure 2. The calculation of DHA follows Equation (7) in terms of percentage recovered (%) and Equation (8) in the terms of amount (mg/g) of FAMEs.

(7) $$DHA\;percentage\;(\%\;wt)\;=\;\frac{DHA\;peak\;area}{total\;area}\;\times \;100$$

(8) $$DHA\;content\;(mg/g)\;=\;\frac{Total\;FAMEs\;\left(\frac{mg}{g}\right)\times DHA\%}{100}$$

## 3. Results and Discussion

### 3.1. σ-Surface

**Figure 3:**
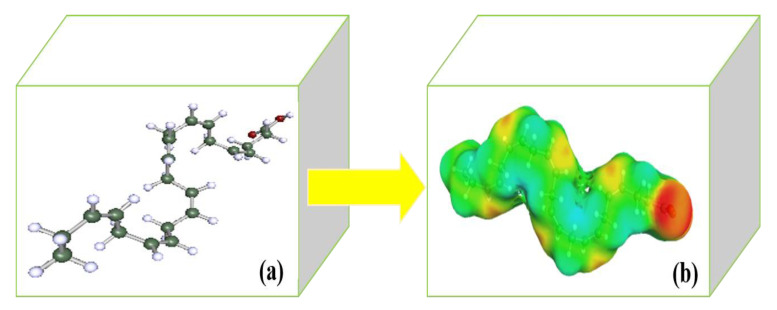
(**a**) Optimized molecular structure of DHA and (**b**) σ-surface of DHA compound performed by COSMOtherm.

The optimized chemical structure and DHA surface were determined using COSMOtherm, and are displayed in Figure 3a,b, respectively. The COSMO-RS 3D screening charge distribution (σ-surface) was used to visualize the hydrogen bonding, lipophilicity/hydrophilicity, polarity and other significant terms related to the qualitative description of DHA molecules.

According to Figure 3b, the surface of DHA is colored in the range of green to red, corresponding to the weakest to strongest polar surface among the molecular structure. The area covered by light blue represents positive polarity (+ve), whereas red indicates a strong negative polar surface (–ve). The σ-surface provides sufficient information for COSMO-RS to estimate the chemical potential of all the compounds.

### 3.2. σ-Potential and σ-Profile

**Figure 4:**
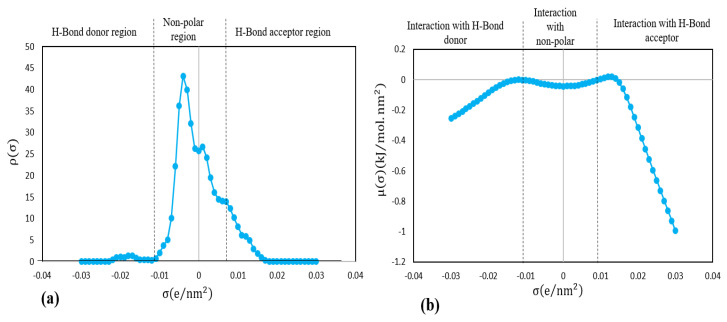
Net surface charge of DHA in terms of (**a**) σ-profiles and (**b**) σ-potential.

The likelihood of distribution for a section of the molecular surface with a particular density of charge is described by the σ-profile. In addition, σ-potential defines a solvent’s probability of interacting with a compound with load density (μ (σ)) and polarity (σ). Since COSMO-RS only needs a compound’s molecular composition to derive its σ-profile, σ-potential and charging density, the instrument can therefore be used to screen any ILs that do not take into account their complexity and unconventional combinations [56]. Figure 4a,b presents the σ-profile and σ-potential of DHA compound derived from COSMO-RS.

The region described as nonpolar in Figure 3a is related to the nonpolar and less polar molecules which are positioned closer to the center of the σ-profile histogram compared to polar molecules [57]. It should be noted that the molecules are considered polar once their screening charge density exceeds ±0.01 e/nm^2^. Furthermore, in the σ-profile, the total calculation time is influenced by the molecular complexity as well as the quality of the initial geometry of a molecule. A greater absolute value of σ shows a stronger compound, i.e., either as a hydrogen donor or acceptor of hydrogen [58]. However, in the σ-potential plot (Figure 4b), which shows the affinity between the mixture components, higher values of positive μ (σ) indicate stronger repulsion between molecules, whereas higher negative values indicate greater intermolecular interactions. For both σ-profile and σ-potential histograms, three main regions were identified and separated with a dashed line. The first region is where the σ values are less than −0.01 e/nm^2^; this is related to the hydrogen bond donor area. The second region is extended between −0.01 e/nm^2^ and +0.01 e/nm^2^; this describes the nonpolarized area. The third region is for σ values higher than +0.01 e/nm^2^ and indicates the hydrogen bond acceptor area. A sequences of sharp peaks can be observed within the nonpolarized area dominated the σ-profile of DHA. The slight peak located at −0.018 e/nm^2^ which corresponds to the hydrogen atoms of DHA molecules indicates the acidic character of DHA and its ability to act as a hydrogen donor, whereas the slight peak at +0.012 e/nm^2^ indicates the hydrogen acceptor characteristic, which is a weak base associated with the carboxylate oxygen atoms of the DHA molecules. To summarize, DHA compounds are able to form nonpolar covalent bonds, as all the main peaks are packed within the nonpolar region, and are therefore, hydrophobic.

### 3.3. Capacity Value

**Figure 5:**
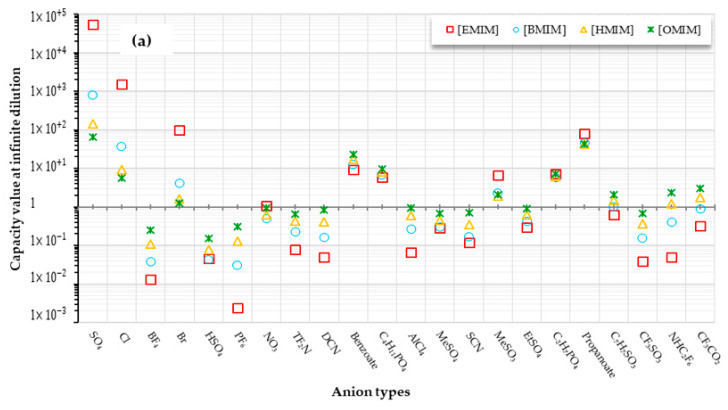

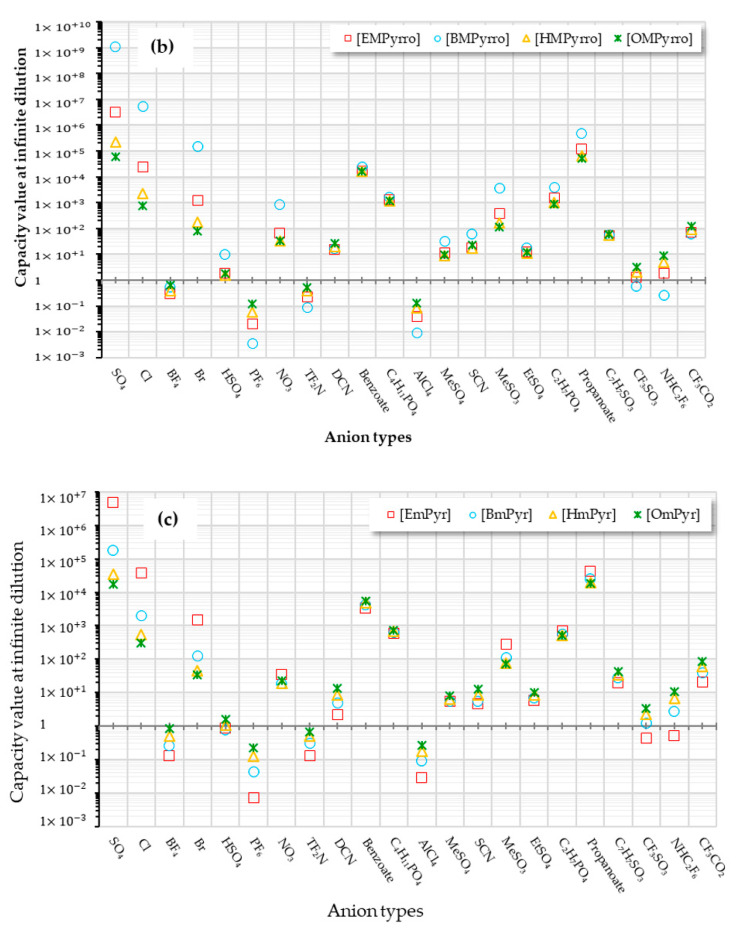

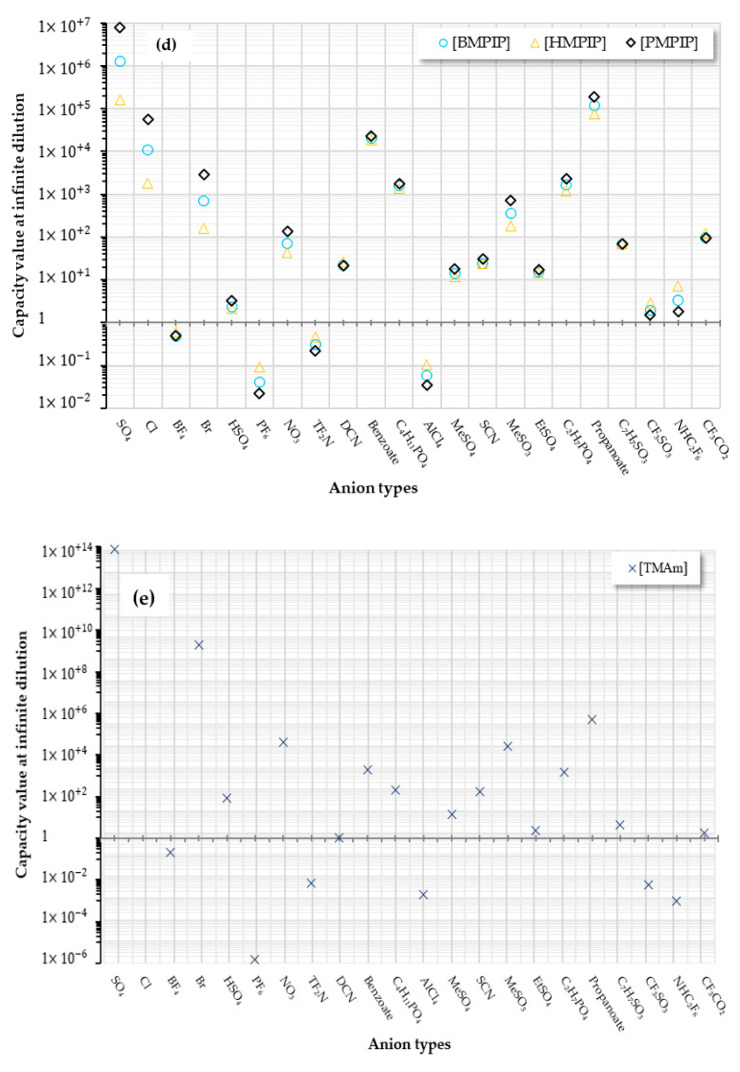
COSMO-RS-predicted infinite dilution capacity values (Y axis) of ILs including: (**a**) Imidazolium-, (**b**) Pyridinium-, (**c**) Pyrrolidinium-, (**d**) Piperidinium- and (**e**) Teramethyl ammonium-based cation alkyl chain lengths with 22 anions (X axis) at 25 °C for DHA extraction.

Figure 5a–e shows the capacity values of cation-based ILs combined with 22 types of anions.

The effects of using different anions, as well as different alkyl chain lengths, on DHA extraction are discussed in further sections.

#### 3.3.1. The Effect of Alkyl Chain Length on the Capacity Value of ILs

In this research, the word “capability value” reflects the interaction between IL molecular structures and the DHA compound. According to Figure 5a–e, capacity values were discovered to vary with cation and anion alkyl chain length; longer cation alkyl chain lengths were followed by reduced IL capacity values, and therefore, considered unfavorable for DHA extraction.

The same trend was generally observed for IL cations based on imidazolium, pyridinium, pyrrolidinium and piperidinium. Instead, the reduction in the cation’s alkyl chain length resulted in greater selectivity, greater variations in the molecular interactions and thus improvements in the suitability of the trainer; however, it also reduced the solubility of DHA in ILs, leading to a reduction in solvent ability [57].

In addition, it was noted that the increase in the length of the alkyl chain adversely affected the extraction ability of the IL. This seems to be true, since the order of capacity is arranged from higher to lower for [EMIM], [BMIM], [HMIM] and [OMIM] accordingly. However, the capacity of IL is significantly reduced based upon the number of anions, as follows: SO_4_^−2^ > Cl^−^> Br^−^> NO_3_^−^. For these exceptions, it may be that the absence of any alkyl chain in the anion results in stronger interactions between anions and cations, thus reducing the interaction with the DHA molecules, which, as a result decreases their extraction capacity [59]. Moreover, as the alkyl chain increases in size, the rise in the length of the alkyl chain leads to a reduction in the load density on the cation, as well as an increase in the molar volume owing to the flexible alkyl chain, which efficiently keeps the ions from packing, and consequently, decreases the density. The motion of IL compounds is also limited as a consequence of enhanced alkyl chain length and the dispersion force increasing as molar volume increases [60].

The effect of inorganic ions on ILs seems to be well recognized by the Hofmeister series. The Hofmeister impact was first researched in 1888 and is observed in a wide spectrum of interfacial phenomena, including protein folding, protein stabilization, colloid behavior, enzyme stabilization and microemulsion composition [61]. However, Hofmeister series are only effected at high salt concentrations, according to a previous report [62]. The electrostatic interaction between the biomolecules and ions is greater than that between the water–biomolecule and the water–ion when the salt concentration is greater than a certain value. Although there are not enough scientific sources to precisely explain the underlying nature of this phenomenon, it can be assumed, based on observation, that ions in solution create a steady interface between hydrophobic and hydrophilic environments when combined with the charged or polar head groups of amphiphilic molecules [63]. In a previous study, the dissimilar effects of anions were observed for different alkyl chain orders, surface potentials and water structures [64]. The research clarified that because of the Hofmeister impact, the ions are able to affect the bulk water structure. The order of anions according to the Hofmeister series were SO_4_^2−^ > Cl^−^ > NO_3_^−^ > Br^−^ > I^−^ > ClO_4_^−^ > SCN^−^. It was shown that ions such as (SO_4_^2−^) can penetrate the head group region of the monolayer, which, and in this case, the DHA molecules [64]. However, in another study, it was pointed out that chaotropic anions were able to penetrate a lipid monolayer [65]. 

In general, the qualitative trend of the ability of anions to induce the salting-out of the ionic liquid closely follows the Hofmeister series. The interpretation of the Hofmeister anion series is not straightforward, but it seems that weakly-hydrated and hydrophobic anions exert a destabilizing effect. The Hofmeister anion series usually quotes [SCN]^−^ as the most destabilizing anion. However, among the widely used anions of ionic liquids, [N(CN)_2_]^−^ is a similarly strong denaturant, and the destabilizing effect of hydrophobic [NTf_2_]^−^ is even greater [66]. The interpretation of the Hofmeister effects at the molecular level is complex, and it is unlikely that the observations can be rationalized by a single or a few molecular properties. Traditional explanations use the concept of kosmotropic (structure-making) and chaotropic (structure-breaking) ions. Even for simple ions, there is, however, little consensus on what these properties actually entail. It is therefore not surprising that the extension to more complex ions is difficult to rationalize in terms of the chaotrope/kosmotrope terminology [67].

In shorter alkyl chains, however, the small anions are easier to replace with the DHA molecule hydroxyl group, although the phenomenon becomes less intense as the length of the alkyl chain increases. As observed from the results, the highest capacities belong to EMIM-based ILs rather than other imidazolium-based types. Moreover, for the 1-alkyl-3-methyl-pyridinium cation, regardless of the anion combination, the increase in the attached alkyl chain length resulted in an increase in extraction capacity. This means that extending the alkyl chain of the IL cation accentuates the steric hindrance on the pyridinium ring. An increase in alkyl chain length leads to an increase in capacity since the intrinsic hydrogen bond within the IL is affected by a larger van der Waals volume; furthermore, the cation–anion interaction decreases and the volume of channel formed between cation and anion increases as a result [68]. This fact was examined in a previous study, and it was observed that an increase in cation alkyl chain length increases the efficiency of alkaloid extraction. However, the adverse effect was observed in cases of sufficiently long aliphatic moieties, e.g., the longest alkyl chain length at the imidazolium cation from hexyl to octyl [69]. An exception was observed with SO_4_^2−^, Cl^−^, Br^−^, propanoate, C_2_H_7_PO_4_ and C_2_H_6_SO_3_. Again, the best capacity was recorded with the short alkyl chain [EMPyr] and inorganic anions rather than organic ones. This can be attributed to the fact that organic molecules are bigger in size, and the Van der Waals exhibits forces between the molecules that impede the exchange of anions. In addition, in past research, imidazolium base ILs were used to separate benzene from a hexane mixture and the selectivity was discovered to have dropped considerably with the increase in the alkyl chain length of the cation. This was attributed to the interaction of hexane with the alkyl group cations. As a result, there was an increase in the alkyl chain length of the cations to significantly decrease the hexane activity coefficient [70].

**Figure 6:**
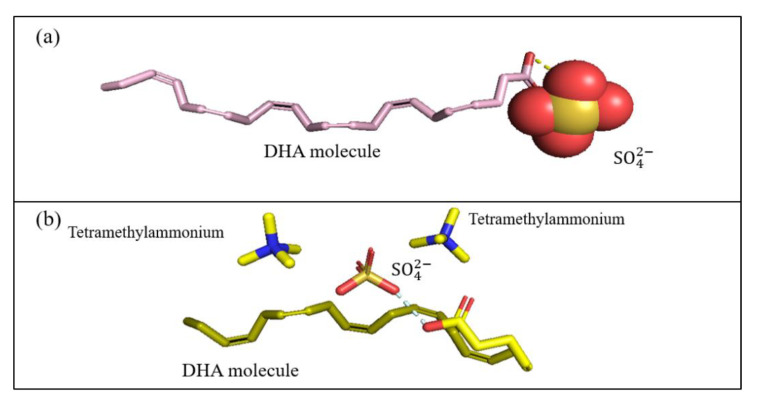

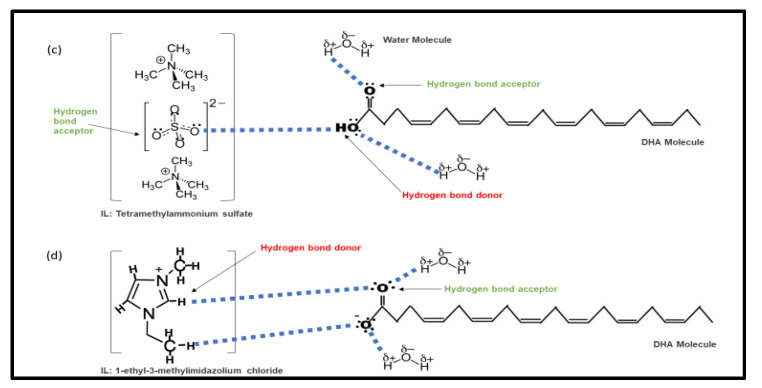
Proposed hydrogen bonding between the IL and DHA; (**a**,**b**) Suggested hydrogen bonding between the SO_4_^2−^ and DHA during extraction, (**c**): DHA and Tetramethyl ammonium sulfate, (**d**) DHA and 1-ethyl-3-methylimidazolium chloride, in aqueous-IL solution.

In the case of the cation 1-alkyl-1-methyl pyrrolidinium, the trend was unique. The extraction capacity increased from ethyl to butyl and showed remarkable fluctuation with increasing alkyl chain length. Based on the obtained results, 1-butyl-1-methyl pyrrolidinium has the highest extraction capacity, especially with SO_4_^2−^, Cl^−^ and Br^−^. The [BMPyrro] was reported to have a very high extraction capacity for the extraction of dibenzothiophene [71] and ethanol from a mixture of hexane and heptane [72], which supports the findings of this study. A similar trend was observed in piperidinium-based ILs, where [BMPIP] cations were a better option compared to [HMPIP] cations in terms of extraction capacity. Likewise, tetramethyl ammonium showed a similar capacity with piperidinium-based ILs. Figure 6 shows an illustration of the hydrogen bond during the extraction process.

Figure 6a,b shows that a H-bond is formed between the sulfate anion and DHA carboxyl group (COOH), while no such bond is formed between the ammonium molecules and DHA. This demonstrates the effect of the anion in the extraction process. The simulation does not neglect the cation effect, where we suggest that the cations are likely contributing to the capacity value rather than the extraction process itself. These observations are supported by the capacity values in Figure 5.

#### 3.3.2. The Effect of Different Anion Types on Capacity Value of ILs

According to the result of Figure 5a–e, sulfate was observed as the best anion used for this study. Although the tetramethyl ammonium and SO_4_^2−^ pair had the highest capacity value, this cannot be concluded from the simulation, as only a single ammonium-based IL was investigated. However, according to the presumption that S=O is a good proton acceptor that can form H-bonds with OH from DHA, it can also be assumed that since the hydrogen bond between the cation and anion (S=O… H) is strong, it will perform well in extraction. This can also be applied for imidazolium-based IL with SO_4_^2−^, as well as with other types of cations with lone pairs of electrons. The distinct patterns in the intensity of the hydrogen bond indicate the possible presence of selective interactions on the imidazolium ring between water, anion and acid protons [73].

In the case of Br^−^ when paired with [EMPyr], a relatively lower capacity value was observed; however, as it paired with [EMPyrro], the values seemed to get higher in the order of SO_4_^2−^ > Cl^−^ > Br^−^ > propanoate. The same manner was observed for [C_n_MPIP] as paired with SO_4_^2−^Cl^−^, Br^−^ and propanoate anions, despite the fact that the cyclic nature of the cation can probably lower the capacity to some extent. Organic anions showed poor capacity towards DHA. This holds true in the cases of C_7_H_7_SO_3_, CF_3_SO_3_, NHC_2_F_6_, CF_3_CO_2_, AlCl_4_, CH_3_SO_4_, SCN and CH_3_CH_2_SO_4_. The exception was observed in C_2_H_7_PO_4_ and propanoate with all short chain cations. 

On the other hand, inorganic anions, namely, SO_4_^2−^, Cl^−^ and Br^−^, could be suitable candidates if combined with short-chain cations such as [EMIM] and [EMPyr]. However, for tetramethyl ammonium-IL, it follows a different behavior, and a larger capacity value was recorded for the anions paired with propanoate. All the electrons of tetramethyl ammonium cations are engaged with methyl groups, and therefore, the compound has a positively charged nitrogen in its structure. Because of the accessibility of the carbonyl group and the hydroxy group, propanoate acts as a useful hydrogen bond donor.

It can be inferred that the anion is in a steadier position due to the larger alkyl groups, and hence, its extraction capacity should be affected accordingly. This is because longer alkyl groups are firmly attached by Van-der Walls forces, which increase with increasing the carbon chain. In this context, X. Li et al. (2016) suggested a correlation between n-3 PUFA extraction and the structure of ionic liquids (aromatic/delocalized cation) as cosolvents for the extraction of PUFA [74]. The hydrophilic and hydrophobic properties of ILs can be determined from the structures of their anions and cations; however; the anion portion is more decisive in determining the miscibility of ILs in water. As shown in [75], ILs derived from [PF_6_]^−^ and [Tf_2_N]^−^ are mostly water-immiscible, and hence, preferred for many IL applications. Particularly, since such ILs are capable of forming biphasic systems, they are considered desirable for use in physical extraction as well. 

In a previous study, two ILs (imidazolium 1-ethyl-3-methylimidazolium ethyl sulfate [EMIM][EtSO_4_] and phosphonium (tetrabutyl phosphonium propanoate [P4444][Prop])) were used as extracting solvents to perform PUFA extraction from *Thraustochytriumsp*. (T18) [76]. According to the results, C22:6n-3 (DHA) was the major component found in all extracts, indicating that T18 was a good source of DHA. Furthermore, the study showed that the extraction was not of high quality and the ILs used were unable to extract the DHA with high selectivity; instead they extracted the total lipids available without oxidizing the unsaturated bonds [76]. This is in agreement with the COSMO-RS results, which showed that propanoate is indeed an excellent option for extraction when combined with ethyl cations.

It can be concluded, however, that increasing the length of the alkyl chain results in reduced selectivity and bad extraction capacity of ILs. Supplementary file (S1) summarizes the results presented in Figure 5a–e for the ILs in order to achieve the highest to the lowest capacity values, and, in contrast, for the infinite dilution activity coefficient. The nonideal characteristics of ILs-DHA mixtures can be better visualized with the aid of an activity coefficient at infinite dilution. Higher values of activity coefficient indicate lower solubility of ILs, and therefore, stronger interaction between solute and IL compounds and vice versa [77,78]. To sum up, one must determine the solubility of compounds in a model solution, as well as the rate of mass transfer, selectivity and the distribution coefficients prior to selecting a suitable IL for extraction [79].

### 3.4. Experimental Validation of COSMO-RS Predicted Extraction Capacity of DHA

**Figure 7:**
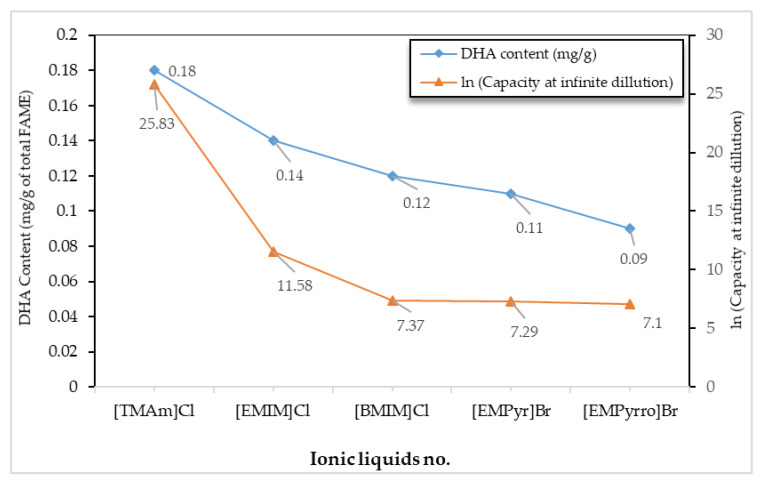
Comparison of the capacity values of selected ILs for the extraction of DHA compounds predicted by COSMO-RS and the experimental DHA extraction yield (mg/g) from *Nannochloropsis sp.* microalgal.

Five types of ILs were selected for a validation study, and a set of experimental evaluations were performed to examine whether COSMO-RS-predicted ILs with high extraction capacities were capable of increasing the extraction of DHA from microalgae. The selected ILs were [EMIM][Cl], [BMIM][Cl], [TMAm][Cl], [EMPyrro][Br] and [EMPyr][Br]. The selection of ILs was based on their high capacity values, as shown in Figure 5 and Supplementary (S1), and their commercial availability. Figure 7 presents the results. It is clear that the extraction yield of DHA is positively correlated with the capacity of the corresponding ILs. The extraction yields, in fact, showed a similar tendency of higher to lower logarithmic values of capacities, i.e., 0.18, 0.14, 0.12, 0.11 and 0.09 (mg/g of total fatty acids), corresponding to [TMAm][Cl], [EMIM][Cl], [BMIM][Cl], [EMPyr][Br] and [EMPyrro][Br], having logarithmic capacity values of 25.83, 11.58, 7.37, 7.29 and 7.10, respectively. It can be concluded that the lower/higher yield of extracted DHA can be determined according to the lower/higher values of COSMO-RS predicted ILs capacities. Thus, the model seemed to be accurate and implementable.

Moreover, the results of DHA extraction in this study were compared to those from a study by [80] which used a standard Bligh and Dyer approach to extract the DHA content from *Nannochloropsis sp.* microalgae. In that study, 0.04 mg/g DHA was extracted using the Bligh and Dyer method. Meanwhile, MAE was employed in a study to determine the fatty acid composition of microalgae at different NaCl concentrations, 10% (*w*/*v*) of solid Loading, 100 °C and for 5 min. The highest DHA yield was reported to be 0.09 mg/g, which is relatively close to value of 0.10 mg/g, obtained through the use of [EMPyrro][Br] in this research.

Overall, the solubility, mass transfer rate and distribution coefficients of all components of the model solution must be considered in order to choose the proper IL for the extraction target.

## 4. Conclusions

To conclude, ILs have been widely studied as green solvents with better performance than conventional solvents for many applications. Finding an appropriate IL with a large number of anions and cations available for extraction is a challenging task, as it involves carrying out many time-consuming and expensive tests. In this study, the COSMO-RS software was chosen for its reliable and fast prediction and understanding of the effect of IL (i.e., cation–anion combinations) structural variations on DHA compound backbones in order to fine-tune ILs for interactions with DHA molecules. According to the results, shorter alkyl chains are preferable. Moreover, it was concluded that inorganic anions are better options than organic ones. Sulfate-, chloride- and bromide-based ILs were shown to have higher extraction capacities. Finally, five selected ILs with the highest predicted extraction capacities were evaluated experimentally, and the DHA extraction yields corresponding to each were obtained and observed to follow a similar trend to the COSMO-RS predicted capacity values. This study provides a method by which to determine suitable IL solvents to extract DHA from microalgae. By using high capacity ILs as solvents, this study proved the viability of obtaining the highest possible extraction yields using a cleaner and more efficient approach; as such, the use of polluting organic solvents may be avoided.

## Figures and Tables

**Table 1:** The chemical structure and formula of the DHA molecule.

Shorthand Sign	Synthetic Name	Trivial Name	Formula	Chemical Structure
C22:6	docosahexaenoic acid	DHA	C_22_H_32_O_2_	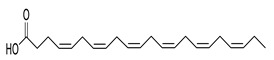

**Table 2:** Classification of anion- and cation-based ILs utilized in this study.

	Anions			Cations	
1	[Cl] ^−^	Chloride	1	[TMAm] ^+^	Tetramethyl ammonium
2	[Br] ^−^	Bromide	2	[EMIM]^+^	1-ethyl-3-methyl imidazolium
3	[BF_4_] ^−^	Tetrafluoroborate	3	[BMIM] ^+^	1-butyl-3-methyl imidazolium
4	[PF_6_] ^−^	Hexafluorophosphate	4	[HMIM] ^+^	1-hexyl-3-methyl imidazolium
5	[NO_3_] ^−^	Nitrate	5	[OMIM] ^+^	1-octyl-3-methyl imidazolium
6	[DCN] ^−^	Dicyanamide	6	[EMPyrro] ^+^	1-ethyl-1-methyl pyrrolidinium
7	[SCN] ^−^	Thiocyanate	7	[BMPyrro] ^+^	1-butyl-1-methyl pyrrolidinium
8	[AlCl_4_] ^−^	Tetrachloro aluminate	8	[HMPyrro] ^+^	1-hexyl-1-methyl pyrrolidinium
9	[C_2_H_7_PO_4_] ^−^	Dimethyl phosphate	9	[MOPyrro] ^+^	1-methyl-1-octyl pyrrolidinium
10	[C_4_H_11_PO_4_] ^−^	Diethyl phosphate	10	[EMPyr] ^+^	1-ethyl-3-methyl pyridinium
11	[C_7_H_5_O_2_] ^−^	Benzoate	11	[BMPyr] ^+^	1-butyl-3-methyl pyridinium
12	[C_2_H_6_SO_3_] ^−^	Methane sulfonate	12	[HMPyr] ^+^	1-hexyl-3-methyl pyridinium
13	[C_7_H_7_SO_3_] ^−^	Toluene-4-Sulfonate	13	[OMPyr] ^+^	1-octhyl-3-methyl pyridinium
14	[CF_3_SO_3_] ^−^	Trifluoro methane-Sulfonate	14	[MPPIP] ^+^	1-methyl-1-propyl piperidinium
15	[SO_4_] ^−^	Sulfate	15	[BMPIP] ^+^	1-butyl-1-methyl piperidinium
16	[HSO_4_] ^−^	Hydrogen sulfate	16	[HMPIP] ^+^	1-hexyl-1-methyl piperidinium
17	[EtSO_4_]^−^	Ethyl sulfate			
18	[MeSO_4_] ^−^	Methyl sulfate			
19	[C_3_H_5_O_2_]^−^	Propanoate			
20	[NHC_2_F_6_] ^−^	Bis(trifluoromethyl)imide			
21	[CF_3_CO_2_] ^−^	Trifluoro acetate			
22	[TF_2_N] ^−^	Bis(trifluoromethylsulfonyl)imide			

## Supplementary Materials

The following are available online at https://www.mdpi.com/2218-273X/10/8/1149/s1, Table S1: Summary of the shortlisted ILs potential for DHA extraction at 298.15 K.
