# Supplementary material for: Prediction of Potential Ionic Liquids (ILs) for the Solid–Liquid Extraction of Docosahexaenoic Acid (DHA) from Microalgae Using COSMO-RS Screening Model
Source: Biomolecules. 2020 Aug 6;10(8):1149. doi: 10.3390/biom10081149 (PMC7464090; doi:10.3390/biom10081149)
Supplement: Supplementary file 1 [file biomolecules-10-01149-s001.pdf]

S1. Summary of the shortlisted ILs potential for DHA extraction at 298.15 K.

Order	Ionic liquids	Infinite dilution capacity value	Infinite dilution activity coefficient value (Ac^{∞})
1	[TMAm]SO_4	7.82E+13	1.28E-14
2	[TMAm]Cl	1.66E+11	6.02E-12
3	[TMAm]Br	1.89E+09	5.29E-10
4	[BMPyrro]SO_4	1.10E+09	9.09E-10
5	[EMIM]SO_4	1.97E+07	5.09E-08
6	[BMPyrro]cl	5.14E+06	1.94E-07
7	[EMPyr]SO_4	4.93E+06	2.03E-07
8	[EMPyrro]SO_4	3.22E+06	3.10E-07
9	[BMPIP]SO_4	1.28E+06	7.83E-07
10	[TMAm]Propanoate	4.97E+05	2.01E-06
11	[BMPyrro]Propanoate	4.84E+05	2.06E-06
12	[HMPyrro]SO_4	2.21E+05	4.52E-06
13	[BMIM]SO_4	1.76E+05	5.68E-06
14	[BMPyr]SO_4	1.75E+05	5.73E-06
15	[HMPIP]SO_4	1.64E+05	6.10E-06
16	[BMPyrro]Br	1.48E+05	6.75E-06
17	[EMPyrro]Propanoate	1.19E+05	8.43E-06
18	[BMPIP]Propanoate	1.17E+05	8.53E-06
19	[EMIM]Cl	1.07E+05	9.31E-06
20	[HMPIP]Propanoate	7.39E+04	1.35E-05
21	[HMPyrro]Propanoate	6.63E+04	1.51E-05
22	[OMPyrro]SO_4	5.90E+04	1.70E-05
23	[OMPyrro]Propanoate	5.20E+04	1.92E-05
24	[TMAm]NO_3	4.24E+04	2.36E-05
25	[EMPyr]Propanoate	4.14E+04	2.41E-05
26	[EmPyr]Cl	3.78E+04	2.64E-05
27	[HmPyr]SO_4	3.53E+04	2.83E-05
28	[TMAm]CH_3SO_3	2.56E+04	3.91E-05
29	[BMPyr]Propanoate	2.48E+04	4.03E-05
30	[HMIM]SO_4	2.35E+04	4.26E-05
31	[EMPyrro]Cl	2.34E+04	4.27E-05
32	[BMPyrro]Benzoate	2.33E+04	4.30E-05
33	[EMIM]Propanoate	2.07E+04	4.82E-05
34	[BMPIP]Benzoate	2.04E+04	4.90E-05
35	[HMPyr]Propanoate	1.95E+04	5.12E-05
36	[HMPIP]Benzoate	1.90E+04	5.26E-05
37	[OMPyr]Propanoate	1.83E+04	5.48E-05
38	[OMPyr]SO_4	1.73E+04	5.79E-05
39	[EMPyrro]Benzoate	1.70E+04	5.87E-05
40	[OMPyrro]Benzoate	1.60E+04	6.23E-05
41	[HMPyrro]Benzoate	1.56E+04	6.40E-05
42	[BMPIP]Cl	1.11E+04	9.05E-05
43	[BMIM]Propanoate	1.00E+04	9.96E-05
44	[OMIM]SO_4	9.16E+03	1.09E-04
45	[HMIM]Propanoate	8.12E+03	1.23E-04
46	[OMIM]Propanoate	7.63E+03	1.31E-04
47	[OMPyr]Benzoate	5.56E+03	1.80E-04
48	[HMPyr]Benzoate	4.81E+03	2.08E-04

49	[BMPyr]Benzoate	4.21E+03	2.38E-04
50	[BMPyrro]$(CH_3)_2PO_4$	3.96E+03	2.53E-04
51	[BMPyrro]CH_3SO_3	3.60E+03	2.77E-04
52	[EMPyr]Benzoate	3.40E+03	2.94E-04
53	[EMIM]Br	2.83E+03	3.53E-04
54	[OMIM]Benzoate	2.30E+03	4.34E-04
55	[HMPyrro]Cl	2.20E+03	4.55E-04
56	[BMPyr]Cl	2.00E+03	5.00E-04
57	[TMAm]Benzoate	1.99E+03	5.02E-04
58	[HMIM]Benzoate	1.88E+03	5.33E-04
59	[HMPIP]Cl	1.84E+03	5.44E-04
60	[BMPyrro]$(CH_3CH_2)_2PO_4$	1.65E+03	6.06E-04
61	[BMPIP]$(CH_3)_2PO_4$	1.65E+03	6.07E-04
62	[BMIM]Cl	1.58E+03	6.32E-04
63	[BMPIP]$(CH_3CH_2)_2PO_4$	1.56E+03	6.43E-04
64	[EMPyrro]$(CH_3)_2PO_4$	1.50E+03	6.67E-04
65	[BMIM]Benzoate	1.47E+03	6.81E-04
66	[TMAm]$(CH_3)_2PO_4$	1.47E+03	6.82E-04
67	[EmPyr]Br	1.46E+03	6.85E-04
68	[HMPIP]$(CH_3CH_2)_2PO_4$	1.41E+03	7.09E-04
69	[EMPyrro]$(CH_3CH_2)_2PO_4$	1.29E+03	7.73E-04
70	[EMPyrro]Br	1.20E+03	8.33E-04
71	[HMPipe]$(CH_3)_2PO_4$	1.18E+03	8.45E-04
72	[EMIM]Benzoate	1.17E+03	8.56E-04
73	[HMPyrro]$(CH_3CH_2)_2PO_4$	1.15E+03	8.68E-04
74	[OMPyrro]$(CH_3CH_2)_2PO_4$	1.13E+03	8.82E-04
